# CRISPR-enhanced microalgae for carbon sequestration and carotenoid production: opportunities and fundamental constraints

**DOI:** 10.1186/s40643-026-01136-y

**Published:** 2026-10-09

**Authors:** Nalinakshan Sreevidya Shruthy, Cheng-Di Dong, Ya-Ting Chen, Shu-Ling Hsieh, Reeta Rani Singhania, Anil Kumar Patel

**Affiliations:** 1 Institute of Aquatic Science and Technology, National Kaohsiung University of Science and Technology, Kaohsiung City, 81157 Taiwan; 2 Department of Marine Environmental Engineering, National Kaohsiung University of Science and Technology, Kaohsiung City, Taiwan; 3 Department of Seafood Science, National Kaohsiung University of Science and Technology, Kaohsiung City, 81157 Taiwan; 4 Centre for Energy and Environmental Sustainability, Lucknow, Uttar Pradesh 226 029 India; 5 Department of Microbiology and Bioinformatics, Atal Bihari Vajpayee Vishwavidyalaya, Bilaspur, Koni, 495009 India

**Keywords:** Microalgae, CRISPR/Cas, Carbon sequestration, Carotenoids, Genetic engineering, Biomass accumulation, Strain improvement

## Abstract

Microalgae represent a promising biological platform for CO_2_ sequestration and the sustainable production of high-value metabolites, particularly carotenoids such as lutein and astaxanthin. However, industrial performance remains constrained by low carbon conversion efficiency, reduced photosynthetic performance, and competing metabolic fluxes that limit product yield. The emergence of CRISPR/Cas-based genome engineering has enabled precise and multiplexed modulation of microalgal metabolic networks, offering new opportunities to overcome these limitations. This review critically evaluates CRISPR-enabled engineering strategies for improving carbon assimilation, photosynthetic efficiency, and carotenoid biosynthesis in microalgae. We discuss advances in transformation and delivery systems across diverse species, CRISPR interference, CRISPR activation, and multiplexed pathway rewiring. Targeted modifications of light-harvesting complexes, carbon concentrating mechanisms, and key carotenoid biosynthetic enzymes are discussed with their reported impacts on biomass accumulation, CO_2_ fixation, and pigment productivity. Emerging evidence indicates that while pathway-specific genetic interventions can significantly enhance metabolic accumulation, these improvements are often not proportionally reflected in whole-cell carbon fixation, revealing underlying constraints imposed by energy allocation, redox balance, and regulatory network coupling. Finally, we highlight the potential of synthetic regulatory circuits and programmable transcriptional control as next-generation tools for dynamic regulation of metabolic fluxes rather than static gene-level modifications. By integrating molecular engineering strategies with physiological and system-level constraints, this review provides a critical framework for developing robust microalgal cell factories for sustainable bioproduction and climate mitigation.

**Graphical abstract:**

**Figure:**
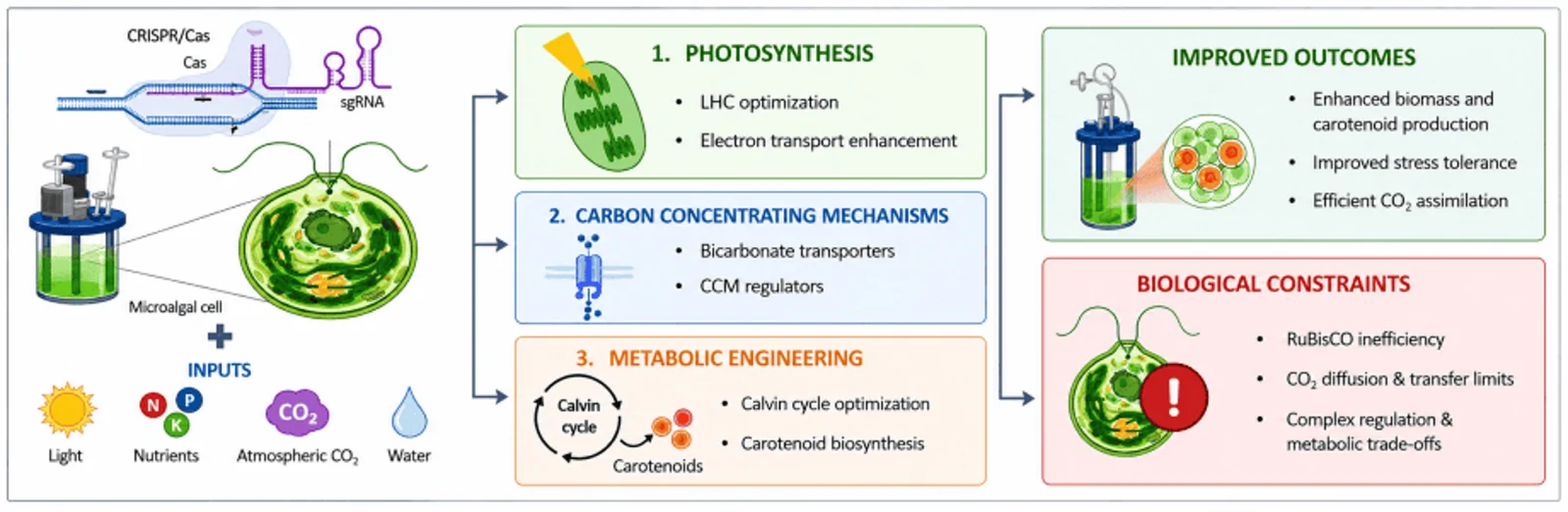

## Highlights

CRISPR improves microalgal CO_2_ fixation and carotenoid biosynthesis.

Multiple CRISPR enables coordinated metabolic pathway engineering.

CCM and photosynthetic pathway are key CRISPR engineering targets.

Carotenoid enhancement often reflects carbon redistribution, not fixation.

Scale-up and transformation efficiency remain major industrial challenges.

## Introduction

Microalgae, a heterogeneous group of photosynthetic organisms found in both terrestrial and aquatic habitats, can directly fix CO_2_ into biomass via photosynthetic carbon assimilation (Zheng et al. 2022; Patel 2025; Patel et al. 2025). They can transform atmospheric CO_2_ into several high-value-added products through effective carbon fixation (Chia et al. 2022). Compared with terrestrial plants, microalgae offer several advantages for carbon sequestration, including rapid growth under optimized light and nutrient conditions (Chen and Hu 2019), the ability to utilize marginal land, and compatibility with controlled cultivation systems such as photobioreactors that enable scalable biomass production (Shareefdeen et al. 2023). In addition, many species naturally accumulate high-value metabolites such as carotenoids and polyunsaturated fatty acids, making them attractive dual-purpose platforms for both carbon capture and bioproduct generation (Xu et al. 2024; Patel et al. 2022; Tambat et al. 2025). Their relatively high photosynthetic efficiency is supported by a pyrenoid-centered CO_2_-concentrating mechanism (CCM), which enhances inorganic carbon fixation under limiting conditions (Wang et al. 2025; Cheng et al. 2025).

Carotenoids are important isoprenoid pigments in microalgae that play a key role in light harvesting and photoprotection. Major carotenoids include β-carotene, lutein, zeaxanthin, fucoxanthin, and astaxanthin, which have considerable applications in the food, nutraceutical, pharmaceutical, and cosmetic industries (Generalić et al. 2023; Mostafa and Hashem 2024). Their biosynthesis is closely linked to photosynthetic carbon metabolism and is influenced by light, nutrient availability, oxidative stress, and cellular energy status (El-Sherbiny and Kalaba 2025). Therefore, improving carotenoid production is an important target for enhancing the biotechnological and commercial potential of microalgae, while maintaining the balance between pigment accumulation and biomass productivity (Sirohi et al. 2022).

CO_2_ fixation and carotenoid biosynthesis are closely connected through intracellular carbon partitioning. Thus, strategies that enhance carotenoid accumulation may alter carbon allocation without proportionally increasing net CO_2_ fixation, creating an important trade-off between carbon assimilation, biomass formation, and metabolite production (Pfleger et al. 2025).

Although microalgae have considerable biotechnological potential, their industrial production remains constrained by low product titers, high capital and operational costs, limited growth rates, contamination risks, and energy-intensive downstream processing (Camacho et al. 2019). As a result, recent engineering strategies increasingly seek to overcome the growth-metabolite trade-off through genetic and metabolic engineering, including CRISPR-mediated genome editing and pathway optimization, to develop stress-resilient strains with improved biomass productivity and pigment accumulation (Sandybayeva et al. 2026).

The emergence of CRISPR/Cas systems has enabled precise and programmable genome editing in diverse biological systems. Its simplicity, high target specificity, and multiplexing capability make it a versatile platform for genome engineering (Lu et al. 2023; Tran et al. 2025). The system relies on a single guide RNA (sgRNA) for target recognition and Cas endonuclease-mediated cleavage. In microalgae, CRISPR tools are applied for targeted gene knockouts and knock-ins, multiplexed genome editing, and transcriptional regulation to generate improved industrial strains (Fajardo et al. 2020). However, its application in microalgae remains limited due to species-specific transformation barriers and limited editing efficiencies. Current research focuses on optimizing editing efficiency, elucidating metabolic regulatory systems, and optimizing strain engineering workflows (Dhokane et al. 2023).

Beyond conventional genome editing through DNA cleavage, CRISPR technology has evolved into a versatile platform for programmable transcriptional regulation. CRISPR interference (CRISPRi) and CRISPR activation (CRISPRa) utilize catalytically inactive Cas9 (dCas9) as a programmable DNA-binding scaffold for reversible gene regulation without permanent DNA cleavage (Kampmann 2018). A single-guide RNA (sgRNA) directs dCas9 to promoter or regulatory regions, where it modulates transcription without inducing DNA double-strand breaks. In CRISPRi, dCas9 alone or fused with transcriptional repressor domains such as KRAB blocks RNA polymerase binding or elongation to suppress gene expression. In contrast, CRISPRa enhances transcription by recruiting transcription activators through direct dCas9 activator fusions like VP64 or scaffold-based systems (Lin et al. 2022). These systems enable reversible, tunable, and multiplex regulation of endogenous genes, making them valuable tools for functional genomics, metabolic engineering, and pathway optimization in microalgae (Li et al. 2026).

This review examines CRISPR-mediated gene knockouts, knock-ins, multiplex editing, and transcriptional regulation for improving CO_2_ fixation and carotenoid production, while also considering related traits such as photosynthetic performance, biomass accumulation, lipid production, and stress tolerance where they influence carbon allocation or strain performance across model and industrially relevant microalgae.

Even though microalgae show strong potential, their efficiency is naturally limited by inherent physiological limitations. Key limitations include the dual carboxylase/oxygenase activity of the enzyme ribulose-1,5-bisphosphate carboxylase/oxygenase (RuBisCO), the energetic burden associated with carbon concentrating mechanisms, and inefficiencies in light energy capture and conversion during photosynthesis (Kupriyanova et al. 2023). Because of these built-in limitations, there is a maximum level of carbon fixation and carotenoid production that cannot be exceeded just by increasing gene expression. This raises a critical question of whether CRISPR-based technologies can overcome these fundamental constraints or primarily enhance performance within existing physiological boundaries. By integrating genetic engineering techniques with reported improvements in physiological traits, this review provides a structured framework to facilitate the systematic development of microalgal platforms for effective carbon sequestration and improved production of high-value-added products.

## Overview of CRISPR technology in microalgae

The emergence of precise modification in living organisms has opened a new direction for understanding complex biological mechanisms and identifying key genes and pathways. Following the discovery of restriction enzymes, continuous studies have been done to establish prokaryotic-derived genetic tools for advanced genome editing technologies, especially the Clustered Regularly Interspaced Short Palindromic Repeats (CRISPR) system (Bhatia et al. 2023). The CRISPR-Cas machinery includes the CRISPR-associated Cas proteins, which function as an adaptive immune system in archaea and bacteria, protecting from viruses, phages, and other invading genetic elements (Liao et al. 2024). This CRISPR system consists of a set of direct repeat sequences that are separated by short spacer regions. These spacer regions are transcribed into CRISPR RNAs (crRNAs), which join with Cas proteins to form a ribonucleoprotein complex after transcription and processing (Dhokane et al. 2023).

**Fig. 1:**
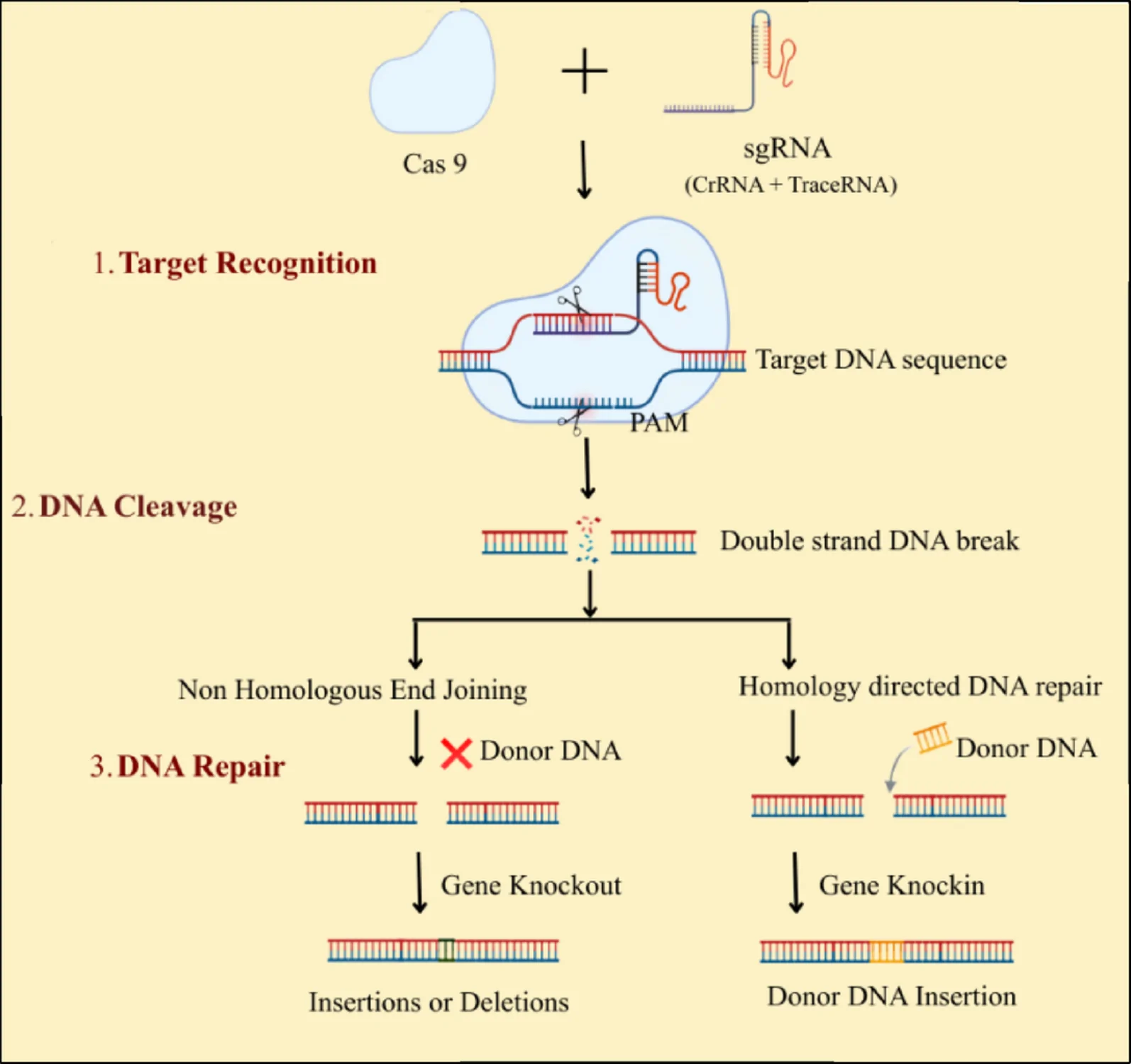
Mechanism of CRISPR/Cas9-mediated DNA cleavage. The CRISPR system consists of 2 main components: the Cas9 endonuclease and Single Guide RNA (sgRNA), which comprises CRISPR RNA (crRNA) and trans-activating CRISPR RNA (tracrRNA). 1. Target Recognition: The Cas-guide RNA complex recognizes the target sequence by PAM-dependent DNA scanning, in which the guide RNA binds to the complementary target sequence, leading to local unwinding and R-loop formation. 2. DNA Cleavage: Successful DNA-RNA hybridization activates the Cas nuclease domain and results in site-specific DNA cleavage. 3. DNA Repair: The induced break is repaired by either Non-Homologous End Joining (NHEJ), an error-prone repair mechanism in which small insertions or deletions occur, or via Homology-directed Repair (HDR), which enables the insertion of desired DNA and results in precise sequence modification

The CRISPR/Cas9 system enables targeted genome editing through sgRNA-mediated recognition of a specific DNA sequence, followed by Cas9-mediated DNA cleavage and repair through non-homologous end joining (NHEJ) or homology-directed repair (HDR) (Fig. 1).

### Development and delivery of CRISPR tools in microalgae

Reliable delivery techniques are crucial for the efficient use of the CRISPR system in microalgae. Hence, an appropriate transformation method and delivery vector are essential to ensure a successful introduction of foreign DNA to an algal cell. The following methods are the most researched approaches used for successful genome editing in microalgae: electroporation, glass bead agitation, particle bombardment, *Agrobacterium*-mediated transformation, and nanoparticle-based delivery (Feng et al. 2023).

Electroporation is the widely used transformation technique for many algal species, particularly in *Chlamydomonas reinhardtii* (Ng et al. 2017)*.* This technique uses electrical impulses to create transient holes in the cell membrane through which the foreign molecules (such as DNA, RNA, or proteins) are introduced (Chen and Hu 2019; Gutiérrez and Lauersen 2021). Nomura et al. (2019, 2020) used the electroporation method to successfully introduce Cas9 ribonucleoprotein complexes into *Euglena gracilis,* resulting in up to 90.1% mutation efficiency in the *EgGSL2* gene through the NHEJ pathway.

Another technique is glass bead-mediated transformation, in which the algae cells, polyethylene glycol (PEG), and foreign genetic material are vortexed to assist the DNA uptake. This method is frequently used for *Chlamydomonas reinhardtii* and certain related species (Economou et al. 2014; Sproles et al. 2021; Gutiérrez and Lauersen 2021).

Microparticle bombardment (biolistic) is a highly versatile gene delivery method that can deliver genetic material into cells with intact cell walls as well as the nucleus, chloroplast, or mitochondria (Li and Bock 2018). In *Phaeodactylum tricornutum*, the CRISPR/Cas9 ribonucleoprotein complexes were transformed through the biolistic method (Serif et al. 2018; Gutiérrez and Lauersen 2021).

*Agrobacterium tumefaciens-mediated* transformation of plant cells utilizes the natural DNA transfer mechanism of *Agrobacterium tumefaciens*, which is responsible for crown gall formation in plants. Successful transformations have been reported in *Chlorella* sp., *Dunaliella bardawil*, *Nannochloropsis* sp., and *Parachlorella kessleri* (Anila et al. 2011; Jennifer and Emmanuelle 2012; Rathod et al. 2013).

Apart from these transformation approaches, another strategy involves the direct introduction of a preassembled Cas9 protein and guide RNA complex into cells as ribonucleoproteins (RNPs). (Chen 2016; Seki and Rutz 2018). In a study, the targeting efficiency of the CRISPR/Cas9 system in *Chlamydomonas* was enhanced by delivering preassembled Cas9-sgRNA ribonucleoprotein (RNP) complexes, a method previously demonstrated to be effective in various other organisms, thereby enabling transient, transgene-free genome editing (Shin et al. 2016; Kim et al. 2014).

Plasmid-based and ribonucleoprotein (RNP)-mediated CRISPR delivery offer distinct advantages for genome engineering in microalgae. Plasmid-based systems introduce DNA encoding the Cas nuclease and guide RNA, enabling sustained intracellular expression and facilitating selection and recovery of edited cells. However, plasmid delivery can result in random integration of exogenous DNA, transgene persistence or silencing, and prolonged nuclease expression, which may increase regulatory concerns when non-GM or marker-free strains are desired (Nomura et al. 2019). In contrast, RNP delivery introduces preassembled Cas protein-guide RNA complexes directly into cells, resulting in transient nuclease activity without introducing a DNA vector. This approach therefore provides an attractive route for transgene-free and marker-free genome editing and may reduce prolonged nuclease exposure and the potential for off-target activity (Jeon et al. 2017). Successful RNP- mediated editing has been demonstrated in microalgae, including *Chlamydomonas reinhardtii*, *Phaeodactylum tricornutum,* and *Euglena gracilis,* although editing efficiency remains highly dependent on the species, target locus, delivery method, and editing strategy. For example, biolistic delivery of Cas9-RNPs in *P. tricornutum* achieved up to 100% editing for specific multiplex knockout combinations, whereas lower efficiencies were initially reported in *C. reinhardtii* (Shin et al. 2016; Serif et al. 2018). Thus, plasmid delivery may be preferable for stable genetic manipulation, whereas RNP delivery is particularly attractive for transient, marker-free editing and applications where foreign-DNA integration is undesirable. The choice should therefore consider species-specific transformation efficiency, editing objectives, cultivation purpose, throughput, and regulatory requirements.

The performance of plasmid-based CRISPR systems also depends on the compatibility of Cas nuclease and guide RNA expression elements with the host species. Species-specific or endogenous promoters are therefore often selected to achieve sufficient and stable expression of CRISPR components, while promoter activity can vary substantially among microalgal lineages. Consequently, Cas variant selection, PAM availability, promoter compatibility, and delivery format should be considered together when developing CRISPR systems for different microalgal species (Jeon et al. 2017; Tran et al. 2025).

Table 1 summarizes the major DNA delivery and genome-editing strategies employed in microalgae, highlighting their transformation efficiencies, precision, advantages, and key limitations. In the final analysis, there are significant differences in the effectiveness of the gene delivery technique between microalgal species. For *C. reinhardtii,* electroporation is well established; its use in species like diatoms and *Nannochloropsis* is limited by their stiff cell walls, which result in low transformation efficiency and significant cell mortality. While the biolistic delivery method has a wide range of species compatibility, it sometimes causes multiple insertions and gene silencing. This makes it difficult to produce a stable strain that consistently expresses the desired gene (Yang and Lu 2025).

**Table 1:** Overview of conventional and CRISPR-based transformation methods in microalgae, with emphasis on transformation efficiency, targeting precision, and major advantages and disadvantages

Category	Transformation method	Representative organism	Reported efficiency	Advantages	Limitations	References
Physical and Chemical DNA delivery	Electroporation	*Chlamydomonas reinhardtii*, *Euglena gracilis, Nannochloropsis* spp.	~ 0.1–5%	Simple setup Applicable to diverse species	Low uptake efficiency High cell damage	Suttangkakul et al. (2019)
	Gene gun (Biolistics)	*Phaeodactylum tricornutum, Chlamydomonas reinhardtii*	~ 0.01–1%	Bypasses cell wall barriers	Multiple insertions Inconsistent expression	Doron et al. (2016)
	PEG/ CaCl_2_-mediated uptake	*Chlamydomonas reinhardtii, Chlorella* spp.	~ 0.05–2%	Mild on cells No specialized equipment	Very low success frequency	Fajardo et al. (2020)
	Agrobacterium-assisted transfer	*Chlorella spp, Dunaliella bardawil, Nannochloropsis spp, Parchlorella kessleri*	~ 1–10%	Higher stability than physical methods	Host specificity constraints	Fajardo et al. (2020)
CRISPR-derived Technology	CRISPR/Cas9 (DNA vector delivery)	*Chlamydomonas reinhardtii, Chlorella* spp.*, Nannochloropsis* spp.	~ 30–80%	Rapid guide design Cost-effective	Off-target risk PAM dependency	Zhang et al. (2019); Patel et al. (2019)
	CRISPR/Cas9 (RNP delivery)	*Chlamydomonas reinhardtii*, *Phaeodactylum tricornutum, Euglena gracilis*	~ 40–100%	Transgene free Reduced off-target activity	Protein instability Delivery challenges	Baek et al. (2018); Poliner et al. (2018)
	CRISPR/Cas12a (Cpf1)	*Chlamydomonas reinhardtii*, *Nannochloropsis* spp.	~ 25–70%	Expanded PAM options Cleaner cuts	Often less efficient than Cas9	(Zetsche et al. 2015; Kim et al. 2016)

### Biological barriers to CRISPR in microalgae

Several significant biological barriers limit CRISPR application in microalgae, with the most critical being that organelles are largely inaccessible to conventional CRISPR delivery systems. This impermeability creates delivery challenges since CRISPR requires guide RNA, template DNA for donor insertion, and Cas9 endonuclease protein to function effectively in organelles (Son and Park 2022).

Beyond these delivery constraints, the potential benefits of CRISPR-based engineering to improve biomass and carbon fixation are also influenced by the inherent physiological limitations of photosynthesis. Photosynthesis naturally converts solar energy into biomass with relatively low efficiency. Terrestrial plants typically capture only about 0.1–2% of the sunlight they receive (Melis 2009). Compared with higher plants, microalgae show much better solar energy conversion potential, with estimated efficiencies of 8–10% and a theoretical biomass conversion productivity of up to 77 g m^−2^ day^−1^. However, under realistic cultivation conditions, their actual photosynthetic efficiency is generally closer to 4.5% (Formighieri et al. 2012; Hu et al. 2023a, b). These physiological constraints are particularly relevant when CRISPR is used to engineer photosynthetic organelles, where efficient delivery and expression of editing components present additional challenges.

*Haematococcus pluvialis,* a commercially important natural producer of astaxanthin, is particularly recalcitrant to genetic manipulation because of its distinctive life cycle and cell-wall characteristics (Kim et al. 2026). Under favorable conditions, cells occur as motile biflagellate vegetative cells, whereas stress induces encystment into non-motile aplanospores with a markedly thick and mannose-rich cell wall that presents a major barrier to intracellular delivery (Guo et al. 2021). Its slow proliferation further restricts suitable transformation windows. Although stable nuclear transformation has been achieved, foreign DNA predominantly undergoes random integration, while efficient homologous recombination has not been established, limiting precise genome editing. Steinbrenner et al. reported herbicide-resistant PDS mutants at approximately 10^–6^ transformants/µg DNA, while Yuan et al. achieved transformation frequencies of approximately 0.4–3 × 10^–5^ transformants/µg DNA using a tubulin promoter vector and motile cell cultures (Steinbrenner and Sandmann 2006; Yuan et al. 2019). Electroporation of intact cells is constrained by the thick cell wall; however, Savvidou et al. (2020) developed enzymatically generated *H. pluvialis* protoplasts and demonstrated their electroporation-mediated uptake of magnetic nanoparticles, supporting protoplast-based approaches for intracellular delivery (Savvidou et al. 2020). PEG-mediated transformation following enzymatic digestion of the cell wall enabled stable ble/EGFP reporter integration in *H. pluvialis* (Guo et al. 2022). However, peer-reviewed evidence for CRISPR/Cas mediated genome editing in *H. pluvialis* remains limited. Although Cas9 RNP delivery by gene gun bombardment has been described in a recent patent, independent experimental validation in a peer-reviewed journal is still lacking (Deng et al. 2024). Future efforts should focus on stage-specific delivery using motile cells, controlled cell-wall permeabilization or protoplast-based approaches, optimization of CRISPR component delivery, and enhancement of precise DNA repair to establish efficient and reproducible genome-editing platforms for industrially relevant strains.

Marine microalgae, due to their unique membrane adaptations, often require higher electric field strengths during electroporation. In contrast, halophilic species such as *Dunaliella* require specialized buffer systems that remain stable under high-salinity conditions. Green algae possessing cellulose-based cell walls typically respond well to enzymatic pretreatment or protoplast-based approaches. Meanwhile, diatoms, which are characterized by rigid silicate frustules, are more effectively transformed using techniques such as particle bombardment or nanoparticle-mediated delivery, which can penetrate their cell structures (Son and Park 2022). Overall, these intrinsic biological constraints continue to limit CRISPR efficiency in microalgae, highlighting the need for species-specific optimization strategies.

The relevance of these delivery barriers depends on the cellular location of the target genes. For carotenoid engineering, many biosynthetic genes are nuclear-encoded, making efficient nuclear transformation particularly important, whereas chloroplast-targeted engineering is more relevant for targets encoded or functioning within the photosynthetic organelle (Ahn and Choi 2026). Thus, species-specific transformation efficiency and successful delivery to the appropriate cellular compartments are important considerations when selecting CRISPR strategies for improving carbon assimilation and carotenoid production.

### Chloroplast -targeted CRISPR genome editing in microalgae

Chloroplast-targeted genome engineering is important for improving photosynthesis, carbon fixation, and pigment metabolism because several proteins involved in these processes are encoded by or function within the chloroplast (Fu and Zhang 2026). For nuclear-encoded CRISPR components, chloroplast transit peptides (cTPs) direct Cas proteins into the chloroplast. Effective cTPs generally contain hydroxylated and positively charged residues, few acidic residues, and lack strongly hydrophobic regions characteristic of secretory signal peptides. Using cTPs derived from endogenous chloroplast-targeted proteins may improve targeting efficiency because chloroplast-targeting signals exhibit species-specific characteristics. Recent experimental work in *Dunaliella salina* demonstrated that endogenous cTPs can effectively direct heterologous proteins to the chloroplast and that sequence extensions beyond the predicted cleavage site can further influence targeting efficiency and accuracy. Notably, an LHCB1-derived cTP enhanced heterologous astaxanthin production by 32.9%, highlighting the importance of species-specific cTP selection and optimization for chloroplast engineering (Dai et al. 2026). However, Cas targeting alone is insufficient because guide RNA delivery or expression within the chloroplast is also required (Carrera-Pacheco et al. 2023).

In chloroplast engineering, Cas9 must be codon-optimized for chloroplast expression, while organelle-specific promoters are required for Cas9 and guide RNA expression. Unlike nuclear genomes, chloroplasts generally lack canonical NHEJ, making precise editing largely dependent on homologous recombination (Yoo et al. 2020). Guide RNA activity can also vary between target sites, while chloroplast-replicating “edit plasmids” containing CRISPR components have been developed to improve organelle-specific expression (Dorogova and Sidorchuk 2023).

Chloroplast genome editing has been most extensively investigated in *Chlamydomonas reinhardtii,* where plastome editing has been demonstrated using CRISPR/Cas systems (Yoo et al. 2020; Nievergelt 2025). In contrast, direct plastid genome editing remains limited in other industrially relevant microalgae. In diatoms such as *Phaeodactylum tricornutum*, CRISPR has primarily targeted nuclear genes encoding chloroplast-localized proteins rather than the plastid genome itself. Multiple plastid genome copies, dependence on homologous recombination, species-specific codon and promoter requirements, and difficulties in multiplex expression further constrain plastid CRISPR engineering.

## CRISPR- mediated gene knockout strategies for enhanced CO_2_ sequestration

Improving carbon fixation efficiency is essential for optimizing carbon capture and utilization (Yang and Lu 2025). Recent developments in CRISPR-based tools have made it possible to modulate important enzymes like RuBisCO and its activase, improve the CCM, and reroute carbon flux toward desired pathways (Priyadarshi et al. 2024; Arshad et al. 2025). Moreover, these technologies enable the engineering of an alternative carbon-utilization pathway by modifying transporter specificity and boosting metabolic enzyme expression. Building on these developments, the following strategies focus on reducing carbon losses, strengthening section regulation, and improving carbon fixation. Table 2 lists the major engineering objectives for microalgae and their corresponding CRISPR-based strategies.

**Table 2:** Summary of CRISPR- mediated metabolic and regulatory engineering strategies used to enhance lipid production, high-value metabolites, carbon capture, stress tolerance, and photosynthetic efficiency in microalgae

Engineering objective	CRISPR- based approaches	Engineering strategy	Key achievements	References
Lipid and biofuel enhancement	Multiplexed CRISPRi/CRISPRa, base editing, biosensor-guided editing	Inhibit starch biosynthesis Engineer thioesterase for tailored fatty acids profile Trigger lipid accumulation under nitrogen limitation	>25% increase in lipid content in *Nannochloropsis* Enrichment of high-oleic acid lipids	Dhokane et al. (2023); Jiang et al. (2025); Yang and Lu (2025)
High-value compound production	CRISPRa, base editing, epigenome editing, prime editing (PE)	Activate carotenoid biosynthesis (e.g., PSY, LCYB) while down-regulating chlorophyll Optimize desaturase enzymes for PUFA production Incorporate heterologous metabolic pathways via PE	Stable Astaxanthin production Improved EPA/DHA accumulation Precise, scarless protein expression	Kuo et al. (2022); Hu et al. (2023a); Yang and Lu (2025)
Carbon capture and utilization	Base editing, multiplexed CRISPRi, and synthetic logic circuits	Engineer RuBisCO for improved carbon fixation Repress TCA cycle flux while activating lipid synthesis under nitrogen-limiting conditions Introducing xylose utilization pathways	Increased carbon flux toward desired products Expanded substrate utilization	Diao et al. (2020); Ambily et al. (2025); Yang and Lu (2025)
Stress tolerance	CRISPRa, base editing, genome-wide screening	Overexpress heat shock proteins (HSPs) and antioxidants Engineer membrane desaturases to enhance cold tolerance Perform CRISPRi screens to identify novel stress-regulatory genes	Enhanced thermotolerance Reduced reactive oxygen species (ROS) Discovery of new resilience genes	Naduthodi et al. (2019); Diao et al. (2020); Yang and Lu (2025)
Boost photosynthesis and biomass	CRISPRi base editing, multiplexed CRISPR	Modulate light-harvesting complex (LHC) genes to reduce antenna size Fine-tune CCMs via targeted point mutations Suppress photorespiration pathways	Enhanced light penetration ~ 10% increase in CO_2_ fixation Decreased carbon loss	Ng et al. (2020); Kumar et al. (2020); Yang and Lu (2025)

### Targeting photosynthetic inefficiencies and carbon loss pathways

Photosynthetic efficiency is relatively low in both terrestrial plants and microalgae. During the initial stages of light harvesting, only a portion of the light spectrum is utilized, and up to 75% of the solar energy is lost by reflection, transmission, or dissipated as heat (Cardona et al. 2018). However, part of this energy loss is necessary for photoprotection; there are many ways to improve the efficiency of light reactions, which could substantially enhance overall biomass and crop productivity (Kupriyanova et al. 2023).

More than half of the energy loss during the conversion of solar energy into chemical energy in photosynthesis results from kinetic imbalances between the rapid photon capture by light-harvesting complexes and the comparatively slower downstream electron transfer processes. One effective approach to enhance energy conversion efficiency and overall photosynthetic performance involves optimizing light utilization by reducing the size of the chlorophyll antenna (Hu et al. 2023b). In *Chlamydomonas reinhardtii*, mutations in the genes that encode components of the chloroplast-localized signal recognition particle (CpSRP), namely *tla3* and *tla4*, were associated with improved solar energy utilization and photosynthetic productivity, especially under high light conditions in mass culture (Jeong et al. 2018; Baek et al. 2018). Recently, the CRISPR-Cas9 system has proven to be an effective method for generating such *tla* mutant cells (Bujaldon et al. 2017). Truncated antenna mutants of *Chlorella sorokiniana* displayed a 30–68% increase in biomass yield within dense cultures representative of industrial photobioreactor conditions, demonstrating improved photon utilization efficiency and greater productivity compared to the wild type (Dall’Osto et al. 2019). Likewise, in the marine diatom *Phaeodactylum tricornutum,* disruption of the *ALB3b* gene, responsible for regulating fucoxanthin-chlorophyll a/c complex synthesis, resulted in a truncated light-harvesting antenna phenotype characterized by reduced photosynthetic pigment content (Nymark et al. 2019).

In *Chlamydomonas reinhardtii*, inhibition of the carbon partitioning gene *PEPC1* led to improved lipid accumulation (893 mg/L at 35 °C) and biomass (2.56 g/L) (Lin and Ng 2023). In *Chlorella sorokiniana*, ASGARD- based CRISPRi using a guanine-rich 20-G sgRNA (sgHG) and dCas9-KRAB induced random transcriptional regulation rather than targeting a defined metabolic gene, with the best transformation reaching 68% protein content (g/g DCW) and 252 mg L^−1^ lipid production (Lin et al. 2022). Similarly, CRISPR-based activation of the *g1248* gene, linked to DNA and mRNA methylation, led to an increase in photosynthetic efficiency (Fv/Fm improved by 12%) and growth 23% in *Nannochloropsis oceanica* (Wei et al. 2022).

CRISPR-based technologies are key components of economical large-scale cultivation because they offer diverse and reliable ways to improve photosynthetic efficiency and boost biomass yield. Specifically, CRISPR interference (CRISPRi) allows fine regulation of gene expression, and may enable the activity of photosystem repair mechanisms under stress conditions (Patel et al. 2019). The targeted knockdown of photorespiratory enzymes through CRISPRi reduces carbon loss, while CRISPR activation (CRISPRa) may enhance the engineered photorespiration bypass route. Moreover, base and prime editing enable precise modulation of key carbon flux enzymes, such as phosphoenolpyruvate carboxylase (PEPC), to promote biomass accumulation. These tools can also strengthen photoprotection by modifying NPQ-related proteins to adjust response kinetics, whereas CRISPRa facilitates the upregulation of genes involved in rapid NPQ recovery (Yang and Lu 2025). Similarly, the disruption of the *ALB3b* gene, associated with fucoxanthin-chlorophyll a/c complex assembly in *Phaeodactylum tricornutum*, led to a 30–40% increase in both rETRmax (maximum Relative Electron Transport rate) and the light saturation parameter (Ek), indicating that the mutant cells can more effectively modulate their photosynthetic machinery in response to changing light conditions (Nymark et al. 2019).

In the case of cyanobacteria, it has been shown that expanding the photosystem’s capacity to utilize a wider range of light wavelengths can enhance photosynthetic performance. Although CRISPR-based studies in this area remain limited, several non-CRISPR genetic engineering approaches have demonstrated the potential of modifying photosynthetic electron transport and light-harvesting systems. Although CRISPR shows great promise for improving photosynthesis in microalgae, its application still faces several challenges. The wide diversity among microalgal species makes it difficult to develop common transformation methods and effective selection markers (Jeon et al. 2017). Additionally, lower transformation efficiency in many non-model species, together with off-target effects and natural gene silencing mechanisms, limits the accuracy and effectiveness of CRISPR-based engineering in microalgae (Lee et al. 2023).

### Disruption of the regulatory genes inhibiting CCM

Guard cells in terrestrial plants control stomatal apertures, which respond to external CO_2_ levels through signaling pathways involving carbonic anhydrase and the protein kinase HT1. However, aquatic photosynthetic species, such as microalgae, usually experience CO_2_-limited conditions because CO_2_ diffuses in water about 10,000 times more slowly than in air. To overcome this difficulty, most microalgae have developed a CCM that raises CO_2_ levels near RuBisCO, maintaining effective photosynthesis (Wang et al. 2016). By precisely introducing point mutations into essential components like carbonic anhydrase and bicarbonate transporters, base editing has been used to fine-tune CCMs, modulating inorganic carbon utilization efficiency and adaptability under a variety of industrial circumstances. Moreover, competing carbon sinks can be suppressed via multiplexed CRISPR interference to improve CCM efficiency (Li et al. 2023; Yang and Lu 2025).

The gene Cia5 is essential in mediating cellular responses to restricted inorganic carbon (CO_2_ and HCO_3_^−^), by increasing the activity of the carbon concentrating mechanism. As a transcriptional regulator, Cia5 regulates the expression of many CO_2_- responsive genes, including those that encode carbonic anhydrase (Chen 2016). Previous studies indicate Cia5 (also called CCM1) binds to important regulatory genes to coordinate the expression of CCM-associated genes. It interacts more readily with target promoters because it has two zinc-finger domains. Targeted disruption of Cia5, the master regulator, offers a useful method for assessing its functional contribution to CO_2_ sensing, CCM control, and the general carbon sequestration process (Chen et al. 2017). CRISPR/Cas9 is one of the main ways to disrupt genes. Compared to the previous gene editing methods, this strategy offers greater flexibility and produces more effective targeted mutagenesis (Shin et al. 2016).

Suppressing negative or limiting regulators can improve CO_2_ uptake, as demonstrated by CRISPR- mediated deletion of important regulatory elements of microalgal CCM. Inorganic carbon absorption pathways are re-shaped, and low CO_2_ inducible gene expression is changed when regulators like Cia5/CCM1 and signaling factors like CAS or LCR1 are disrupted (Wang et al. 2016). CRISPR-based interventions in microalgae show context-dependent effects on CO_2_ sequestration depending on the targeted gene. While some studies show that repression of metabolic genes such as PEPC1 can enhance carbon flux toward biomass accumulation, CRISPR/Cas9-mediated knockout of the Cia5 gene in *Chlamydomonas reinhardtii* significantly reduced CO_2_ sequestration efficiency by impairing CCM activation. Specifically, CO_2_ removal efficiency decreased from 27, 37, and 21 in the wild-type strain to 16%, 23%, and 9% in the Cia5 knockout strain under 0.04%, 0.5%, and 1% CO_2,_ respectively. These findings highlight the importance of selecting appropriate genetic targets for improving biological carbon fixation (Asadian et al. 2022).

CCM enhances CO_2_ delivery to RuBisCO, but this improvement comes with an energetic cost because active bicarbonate transport and CO_2_ retention depend on adenosine triphosphate (ATP) or proton motive force. Maintaining elevated intracellular CO_2_ levels requires continuous energy investment to support efficient carboxylation. Modeling studies of pyrenoid-based CCMs estimate an additional energy requirement of approximately 1.3 ATP per CO_2_ fixed, while CO_2_ or HCO_3_^−^ leakage can further increase this demand (Gerotto et al. 2020). Therefore, strengthening CCM activity may divert energy from growth and biomass production, highlighting the need to evaluate ATP trade-offs when engineering CCM pathways (Fei et al. 2022). Collectively, these studies demonstrate that CRISPR-based strategies can enhance specific components of photosynthetic machinery.

### Fundamental biochemical and biophysical limitations of microalgal CO_2_ fixation

Microalgal CO_2_ fixation is constrained by several biochemical and physical factors that reduce the overall photosynthetic efficiency, including the catalytic properties of RuBisCO, cellular energy balance, carbon allocation, and CO_2_ availability. RuBisCO has a relatively low catalytic turnover and significant oxygenase activity, which limits CO_2_ fixation efficiency and increases the energetic cost of carbon assimilation (Cummins 2021; Iñiguez et al. 2019). These characteristics are important when interpreting CRISPR-based engineering outcomes, because increasing RuBisCO abundance or modifying its associated components may improve carbon fixation under specific conditions but cannot necessarily overcome its intrinsic kinetic limitations. Similarly, CCMs increase CO_2_ availability around RuBisCO and favor carboxylation, but active inorganic carbon transport and CO_2_ retention require additional energy, creating a trade-off between enhanced carbon acquisition and cellular energy balance (Gerotto et al. 2020.

To overcome these challenges, many microalgae have evolved CCMs that increase CO_2_ levels around RuBisCO and favor carboxylation. However, these mechanisms require additional energy, highlighting an inherent trade-off between improving carbon fixation efficiency and maintaining cellular energy balance. In the case of some marine photosynthetic organisms, this evolved CCM also enables them to utilize bicarbonate, which effectively bypasses the limitations of low CO_2_ concentrations (Dao et al. 2025). Photorespiration is also one of the major limitations in microalgae. CCMs from different marine species show quite different efficiencies. For example, *Nannochloropsis* has been shown to possess a relatively inefficient CCM (Gee and Niyogi 2017; Dao et al. 2025). Additionally, even species with highly effective CCMs may release notable amounts of glycolate under certain conditions such as during declines in CO_2_ availability in algal blooms or following sudden increases in light intensity (Gee and Niyogi 2017).

## CRISPR-based metabolic strategies for CO_2_ fixation and carotenoid biosynthesis

Considering the Calvin cycle as the major pathway for photosynthetic carbon assimilation, improving regulatory control is crucial for improving CO_2_ fixation efficiency. This cycle, which takes place inside the chloroplast and consists of a coordinated series of events mediated by eleven enzymes, serves as a metabolic core in plants and algae (Yang et al. 2017). The following sections summarize the main knock-in strategies for CO_2_ fixation and carotenoid biosynthesis.

### CO_2_ fixation via overexpression strategies

Microalgae are ideal candidates for sustainable biotechnological production since they naturally synthesize carotenoids efficiently and actively utilize CO_2_ (Koller et al. 2014). Through both genetic engineering and improved cultivation techniques, increased biomass growth not only increases the cellular carotenoid levels but also enhances the total pigment production (Yang et al. 2017). Several approaches have been developed to improve microalgae’s ability to fix carbon. Among them, endogenous gene overexpression and targeted mutagenesis have been used to create RuBisCO variants that exhibit improved catalytic performance by altering proteins that interact with RuBisCO activase or *rbcL* and *rbcS*, the large and the small subunits of RuBisCO. However, increasing RuBisCO or its activase levels does not overcome the enzyme's intrinsic catalytic limitations, and reported improvements in biomass productivity are frequently condition-dependent and not universally scalable. Similarly, increasing RuBisCO levels in *Synechocystis* sp. PCC 6803 has resulted in quantifiable improvements in fatty acid synthesis and photosynthetic efficiency (Eungrasamee et al. 2020, 2021). A RuBisCO variant with decreased enzymatic activity was produced by targeted mutagenesis of the *rbcL* gene. Interestingly, this variant caused *Chlamydomonas reinhardtii* to produce more hydrogen and accumulate enhanced total lipid than the wild-type strain (Esquível et al. 2017). These findings suggest that partial reduction of RuBisCO activity may redirect carbon flux toward alternative metabolic pathways. In addition to RuBisCO, several additional Calvin-Benson cycle enzymes, including fructose-1,6-bisphosphatase (FBPase), fructose-1,7-bisphosphatase (SBPase), and fructose-1,6-bisphosphate aldolase (FBA), serve as important regulatory points for improving photosynthetic efficiency. Collectively, these studies indicate that modifying Calvin cycle enzymes can improve metabolic flux and growth under specific conditions. In several cases, overexpression of the Calvin cycle enzymes has resulted in imbalanced metabolism or reduced growth, suggesting that pathway flux is tightly regulated and sensitive to perturbation. These observations also show that carbon fixation efficiency depends not only on individual enzyme abundance but also on coordinated regulation of pathway flux, energy availability, and redox balance.

### Engineering of carotenoid biosynthesis pathways

**Fig. 2:**
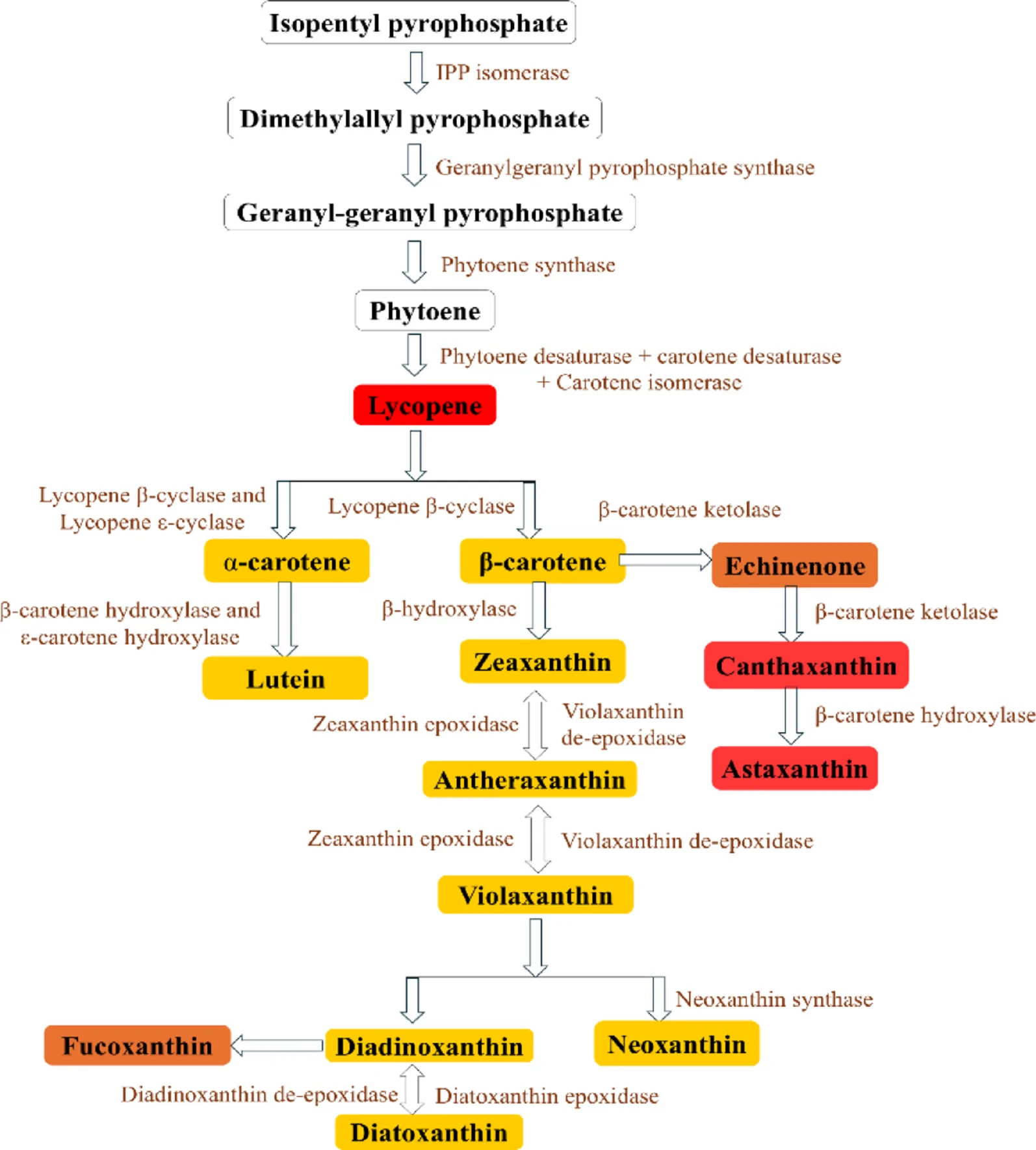
Pathway of carotenoid biosynthesis. Schematic representation of the carotenoid biosynthesis pathway in microalgae, showing the key enzymatic steps leading to the production of major carotenoids

Microalgae are rich sources of various carotenoid pigments, such as lutein, canthaxanthin, astaxanthin, zeaxanthin, and fucoxanthin. Microalgal photosynthesis relies on a diverse suite of pigments, including chlorophylls, carotenoids, and phycobiliproteins, that function in light harvesting and photoprotection. Advances in gene editing and metabolic engineering now provide effective strategies to further enhance pigment biosynthesis in these organisms (Srivastava et al. 2022; Hu et al. 2023b). As illustrated in Fig. 2, carotenoid biosynthesis in microalgae involves a series of enzymatic conversions from isoprenoid precursors to major carotenoids, highlighting potential targets for metabolic engineering.

**Fig. 3:**
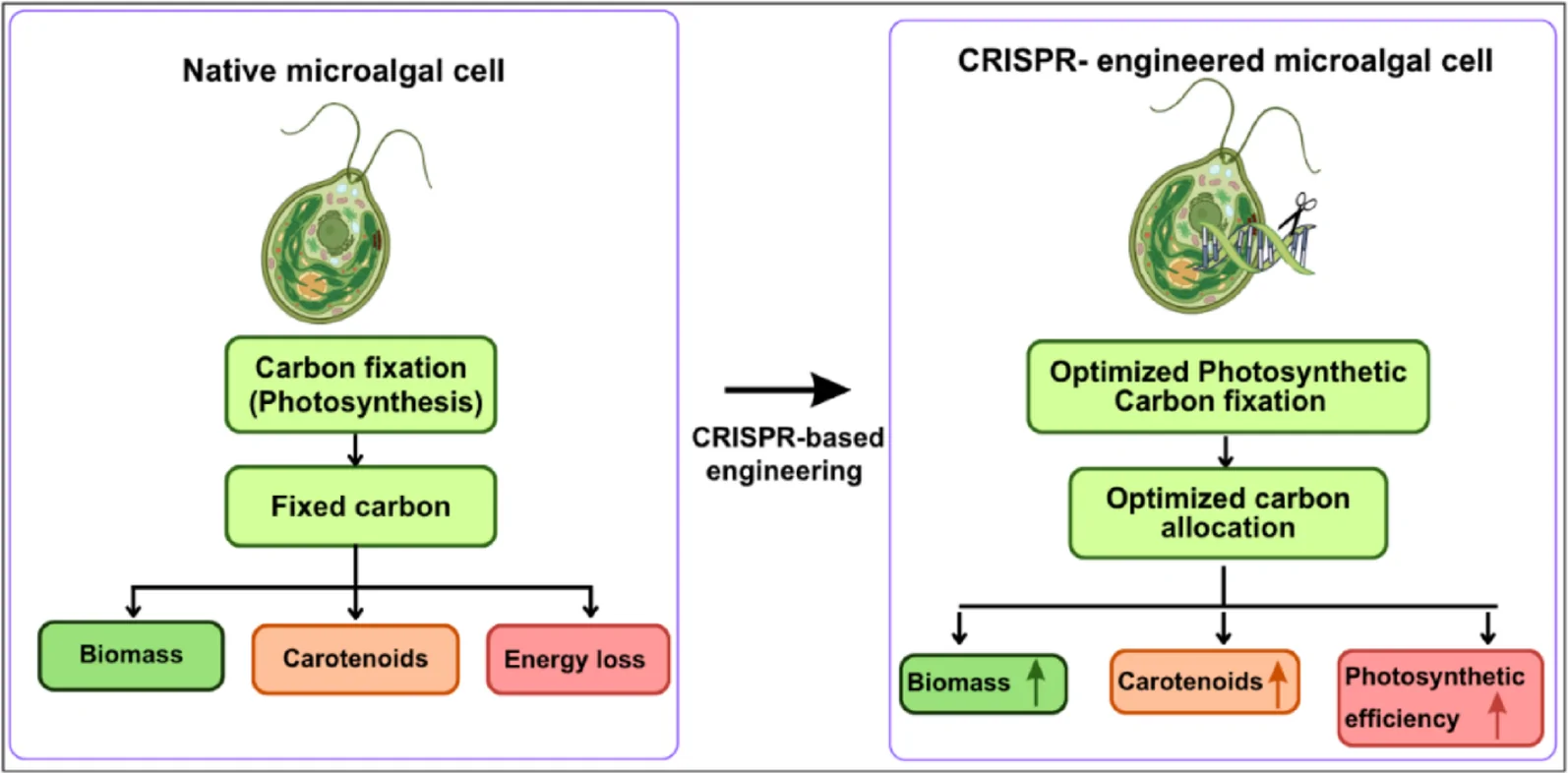
Effects of CRISPR engineering on microalgae performance. Conceptual comparison of native and CRISPR-engineered microalgal cells, illustrating potential improvements in carbon fixation, photosynthetic efficiency, energy utilization, biomass accumulation, and carotenoid production following targeted genetic engineering

A recent analysis has identified the key genes and enzymes involved in carotenoid biosynthesis, and structural models have been generated for three major enzymes, DXR, PSY, and ZDS (Narang et al. 2022). It has been demonstrated that overexpression of the genes encoding DXS and DXR increases lutein and β-carotene production in *Chlamydomonas reinhardtii* by roughly 1.9 and 1.7 times, respectively (Morikawa et al. 2018). Although not directly targeting carotenoid enzymes, transcriptional modulation of regulatory genes can indirectly improve photosynthetic performance and metabolic productivity. A CRISPR/dCas9-based transcriptional activation platform was developed for *Nannochloropsis oceanica* IMET1, in which dCas9 was fused to the VP64 activator to modulate target gene expression effectively. Using this system, transcription of the methyltransferase-related gene g1248, involved in DNA and RNA methylation, was upregulated by approximately 2–6-fold. Mutant strains carrying this activation construct also exhibited notable improvements, showing a 23% increase in growth and a 12% rise in Fv/Fm relative to the wild type under ambient CO_2_ conditions (Wei et al. 2022). In *Dunaliella viridis*, chloroplast integration of modified CRTR-B and *BKT*, which encode two central enzymes for astaxanthin formation in *Haematococcus pluvialis,* led to substantial pigment enhancement under high-light treatment, reaching 77.5 ± 7.7 μg/g DW of astaxanthin and 50.1 ± 0.8 μg/g DW of carotenoid ketolase products (Lin et al. 2019). While these strategies effectively enhance pigment production, they do not directly contribute to increased CO_2_ sequestration capacity, highlighting a conceptual distinction between metabolic engineering for product yield and carbon capture efficiency. Targeted disruption of *ZEP* in *C. reinhardtii* yielded dramatic pigmentation shifts, with zeaxanthin levels increasing 56-fold and productivity rising 47-fold relative to wild type (Baek et al. 2018). Joint knockdown of *LCYE* and *ZEP* redirected carotenoid flux toward the β-carotene/zeaxanthin branch, resulting in approximately 60% higher zeaxanthin content than the ZEP single knockout after 3 days of cultivation (Song et al. 2020). Additionally, under nitrogen constraints, simultaneous CRISPR-Cas9-mediated deletion of *ZEP* and *AGP* in *C. reinhardtii* increased oil and macular pigment formation by over 80% (Song et al. 2022). These improvements are therefore best interpreted as optimization of carbon partitioning. Overall, CRISPR-based knock-in and overexpression strategies have demonstrated the ability to enhance specific metabolic pathways and increase product yields. Consequently, their contribution to carbon sequestration should be interpreted within the broader context of metabolic trade-offs and system-level constraints. Moreover, direct comparisons between studies remain difficult due to differences in cultivation conditions, productivity metrics, and methods used to assess carbon fixation. The conceptual impact of CRISPR-mediated metabolic engineering on carbon allocation, biomass formation, and carotenoid accumulation, while remaining constrained by intrinsic photosynthetic limitations, is illustrated in Fig. 3.

## Multiplex CRISPR strategies for simultaneous pathway engineering

Multiplexed gene knockout and insertions are supported by CRISPR technology, enabling the simultaneous change of many genomic targets. Additionally, by facilitating coordinated gene repression or activation within complex metabolic pathways and regulatory circuits, CRISPRi and CRISPRa systems offer additional transcriptional regulation (Dhokane et al. 2023).

### Multiplexed targeting for coordinated enhancement of CO_2_ fixation and carotenoid biosynthesis

**Fig. 4:**
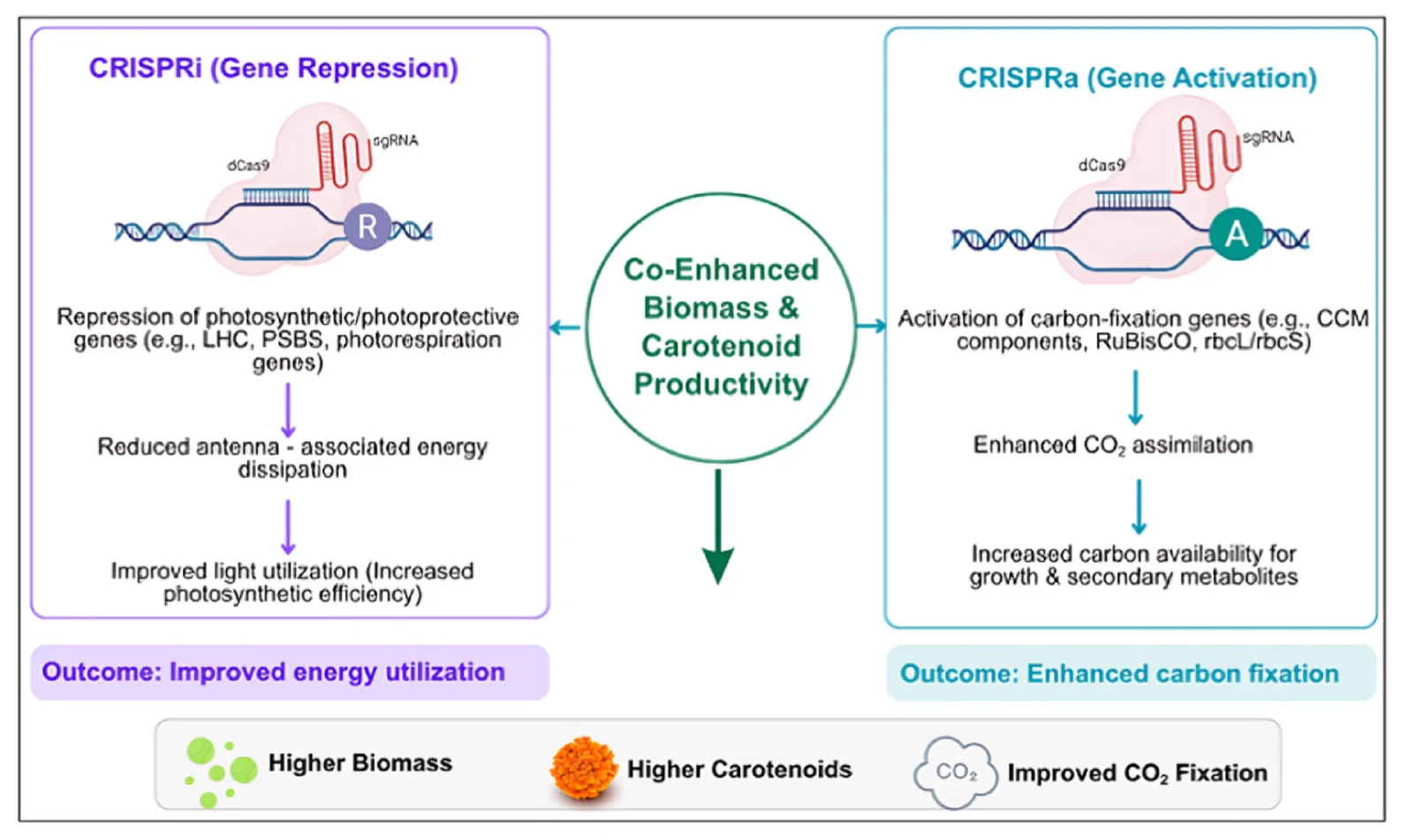
Multiplexed transcriptional regulation in microalgae. Schematic representation of multiplexed transcriptional regulation using catalytically inactive Cas9 (dCas9). On the left, CRISPR interference (CRISPRi) represses photosynthetic antenna and photorespiration-related genes through the repressor domain. It results in reduced antenna size and improved photosynthetic efficiency. On the right, CRISPR activation (CRISPRa) enhances the expression of carbon fixation-associated genes via the activator domain. The simultaneous repression and activation of distinct gene sets may enable coordinated enhancement of carbon fixation, biomass accumulation and carotenoid production

The scope and efficacy of genomic editing and transcriptional regulation have been greatly expanded by multiplexed CRISPR technologies, an advanced method in which multiple guide RNAs (gRNAs) or Cas enzymes are expressed simultaneously (McCarty et al. 2020). Compared to conventional single-target CRISPR uses, this is a significant extension of conventional single-target editing approaches. Multiplexed CRISPR systems employ multiple guide RNAs to simultaneously regulate or edit several genomic loci within the same cell. The construction, production, and processing of synthetic guide RNA arrays *in vivo* are the key innovations (Dong et al. 2018)*.* These arrays are frequently built with RNA processing systems, such as tRNA-based processing systems or ribozyme-based cleavage mechanisms, that can produce several functional gRNAs from a single transcript. While multiplexing increases the scale of genetic intervention, the cumulative effects of multiple edits can introduce metabolic burden, regulatory imbalances, and unintended epistatic interactions, which are rarely systematically evaluated (Campa et al. 2019). Such interactions may reduce cellular robustness and complicate the prediction of engineering outcomes under industrial cultivation conditions. Since the metabolites have significant commercial and biological value, most CRISPR-Cas9 applications in microalgal research concentrate on increasing lipids and carotenoid biosynthesis (Jothibasu et al. 2021; Kossalbayev et al. 2025). Microalgae-derived carotenoids have potent antioxidant properties and have been linked to protection against several diseases and malignancies, including arthritis and cardiovascular conditions. In most cases, what improves is the amount of a specific product, not necessarily the total amount of CO_2_ being fixed by cells. Considering the well-known model microalgae, *Chlamydomonas reinhardtii* and *Phaeodactylum tricornutum* are the most often used species that have been modified with CRISPR-Cas9 to enhance lipid and carotenoid synthesis (Dhaouadi et al. 2020; Celi et al. 2022). Taparia et al. (2022) developed an episomal system based on CRISPR/Cas9 that can produce marker-free genome editing and multiplexed targeting of the *StLDP* gene. Their work produced a streamlined episome expressing Cas9 and multiple sgRNAs targeting different sites within the Stramenopile-type lipid droplet protein (*StLDP*) gene, achieving knockout efficiencies between 6.7 and 13.8%. It demonstrated the feasibility of multiplexed, marker-free editing of a single target gene in diatoms. The multiplexed targeting strategy shown in Fig. 4 highlights how simultaneous CRISPRi-mediated repression and CRISPRa-mediated activation can be integrated to promote photosynthesis and carbon assimilation. When multiple pathways are engineered together, enhancement of one pathway may impose trade-offs such as reduced growth or increased energetic demand.

### Use of CRISPR arrays and polycistronic sgRNAs in microalgae

Achieving the extensive genetic modification required for advanced microalgal metabolic engineering, such as boosting metabolite production or reconstructing complex biosynthetic routes, often demands editing multiple genomic sites at the same time (Kumar et al. 2020; Gupta et al. 2024). Since multiplex CRISPR technologies allow the simultaneous delivery of multiple guide RNAs (gRNAs) that target distinct genes within a single cell, they are ideal for these purposes. Both ribozyme-based architectures that self-cleave from precursor transcripts and polycistronic gRNA constructs, where gRNA is flanked by processing signals, are released by endogenous RNases P and Z, and are common techniques that have shown good performance in diatoms (Kumar et al. 2020; Naduthodi et al. 2021). In practice, this approach does not work equally well in all microalgae, because each species has different genetic and regulatory constraints. Another method uses CRISPR arrays and Cas12a for its inherent multi-crRNA processing activity to enzymatically break a single transcript with multiple crRNAs separated by direct repeats into independent guides (Gong et al. 2021). Another alternative is the orthogonal Cas system, which uses many Cas proteins combined with their gRNAs generated from different vectors or promoters to target different loci individually while minimizing interference (McCarty et al. 2020). Multiplex editing enables rapid metabolic redesign, simultaneous disruption of competing pathways, and coordinated tuning of biosynthetic routes. It also facilitates the integration of beneficial traits, such as enhanced growth, stress resilience, and metabolite accumulation, within a single transformation event. For example, multiplex CRISPRa/i could potentially be used to coordinately regulate key carotenoid biosynthetic genes, including phytoene synthase (PSY), phytoene desaturase (PDS), carotenoid isomerase (CRTISO), β-carotene ketolase (BKT), and β-carotene hydroxylase (CRTR-B), to redirect metabolic flux toward astaxanthin production (Chen et al. 2023; Sun et al. 2025). Overall, multiplex CRISPR is a powerful tool for fine-tuning microalgal metabolism. However, coordinated editing of multiple pathways increases the complexity of metabolic regulation and may generate unpredictable phenotypic interactions.

## CRISPRi and CRISPRa for modulation of gene expression

Although precise genome modification in microalgae was made possible by early CRISPR-Cas tools, primarily Cas9-and Cas12-driven knockouts and knock-ins, the demands of contemporary metabolic engineering extend beyond straightforward DNA cleavage (Sproles et al. 2021; Feng et al. 2023). Instead of permanently disrupting genes, regulatory control is needed to improve features like increased lipid accumulation, enhanced stress tolerance, or redirected metabolic flux. These objectives can be addressed by CRISPR interference (CRISPRi) and CRISPR activation (CRISPRa), which allow for programmable modulation of endogenous gene expression (Ambily et al. 2025; Rau and Ertesvåg, 2021). CRISPRi and CRISPRa allow fine control over gene expression, but their effects are still limited by the cell’s energy capacity and underlying metabolic constraints. These systems support multilayered interventions like coordinated regulation of metabolic pathways, reversible tuning of biosynthetic routes, and precise adjustment of key enzymatic steps essential for complex trait optimization. Microalgae also serve as valuable systems for studying regulatory control of carotenoid biosynthesis because of their physiological and genetic similarities to plant cells. Traditional methods of increasing carotenoid accumulation include either targeted repression or overexpression of important enzyme genes, including PSY, PDS, and BKT (Velmurugan and Kodiveri 2022).

### CRISPR interference for repression of competing carbon utilization pathways

CRISPR interference (CRISPRi) provides a sophisticated method for redirecting Carbon utilization in microalgae by selectively suppressing rival metabolic pathways without causing long-term genetic changes. This Technology is especially useful for rerouting carbon flux into desired biosynthetic routes since it makes use of CRISPR- derived transcriptional modulators that allow for coordinated rewiring of complicated metabolic networks and controllable gene expression (Alerasool et al. 2020). Fine-tuning pathway repression may improve the efficiency of microalgal biomanufacturing platforms by fine-tuning the level of pathway repression and regulating carbon flow without compromising cellular activities, supporting the goal of engineering robust and highly productive microalgae (Yang and Lu 2025). Such improvements are typically pathway-specific and do not necessarily translate into an increase in overall biomass productivity or CO_2_ sequestration efficiency. Kao and Ng (2017) showed how CRISPR interference can be used to reroute carbon usage by effectively reducing flux into a competing metabolic branch by CRISPRi-mediated regulation of the carbon-fixing enzymes PEPC in *Chlamydomonas reinhardtii,* which improved lipid accumulation. Blocking certain pathways can boost product formation, but it may also slow growth or stress the cell. These findings demonstrate the potential of CRISPRi for pathway specific metabolic redirection in microalgae.

### CRISPR activation to upregulate carotenogenic and CCM genes

CRISPR Activation (CRISPRa) is a promising strategy for improving carotenoid production and CCM efficiency in microalgae via targeted transcriptional upregulation. The method is well-suited for simultaneously enhancing carotenogenic pathways and CCM systems since it makes use of CRISPR-derived transcriptional modulators that allow for variable gene expression and the coordinated rewiring of complex metabolic networks (Brocken et al. 2018). To target promoter regions of genes of interest, CRISPRa uses catalytically inactive Cas9 (dCas9) fused to transcriptional activator domains such as VP64, p65, or Rta. CRISPRa-mediated transcriptional activation has been demonstrated in microalgae, including *Chlorella sorokiniana* (Lin et al. 2022), and could potentially be applied to genes involved in carotenoid biosynthesis and CO_2_ fixation. However, enhanced CCM activity also increases energy demand, which can reduce the net benefit. The strategic deployment of CRISPRa can simultaneously enhance photosynthetic efficiency through improved CCM function and increase valuable carotenoid accumulation, supporting the development of robust, high-productivity microalgal cell factories capable of producing both enhanced biomass and high-value pigments for commercial applications (Yang and Lu 2025). Notably, increased carotenoid production reflects enhanced carbon allocation toward secondary metabolism rather than an increase in net CO_2_ fixation. These improvements are usually modest and depend on conditions, showing that they indicate that these approaches primarily fine-tune rather than fundamentally transform cellular metabolism. Wei et al. (2022) established a CRISPR/dCas9- VP64 activation system in *Nannochloropsis oceanica* and achieved ~ 2–6× upregulation of a target gene at the transcriptional level, demonstrating the feasibility of CRISPRa to upregulate endogenous pathways in a marine microalga. Additionally, transcriptional activation efficiency varies significantly across species due to promoter strength, chromatin accessibility, and gene silencing mechanisms, limiting the predictability of CRISPRa-based interventions.

### Synthetic transcriptional circuits for fine-tuned regulation in microalgae

Synthetic transcriptional circuits represent a sophisticated approach for achieving precise, programmable control over gene expression in microalgae through engineered regulatory networks that respond dynamically to cellular and environmental conditions. Transcriptional modulators (CRISPRa/i) and biosensor-integrated logic gates, which offer variable gene expression and dynamic, autonomous regulation in response to environmental stimuli, enable the construction of these circuits using the CRISPR toolkit (Kang et al. 2024). Although promising, these systems are still difficult to implement because of limited knowledge, metabolic burden, and stability issues. Combinations of transcriptional activators, repressors, and inducible promoters that respond to particular inputs like light intensity, nutrient availability, pH, or metabolite concentrations can be used to create these circuits as logic gates (AND, OR, NOT). To produce complex regulatory behavior that enables temporal regulation of metabolic pathways and stress responses, the circuits can include oscillators, toggle switches, and feedback loops (Teixeira and Fussenegger 2024). Synthetic transcriptional circuits may enable autonomous optimization of metabolic flux, stress tolerance, and product formation. Such systems could support the development of robust and highly productive microalgal cell factories (Yang and Lu 2025). These engineered systems also consume extra energy and resources, which are often not measured. Kassaw et al. (2022) developed an episome-based, chemically inducible gene-expression platform in the marine diatom *Phaeodactylum tricornutum*, achieving up to ~ 180-fold and ~ 90-fold dynamic range of induction with β-estradiol and digoxin, respectively, demonstrating a robust, tunable, and reversible synthetic transcriptional circuit for microalgae. However, long term stability and predictable circuit behaviour under industrial cultivation conditions remain major challenges.

## Recent advances and experimental studies

Currently, most industrial carotenoids are obtained either through chemical synthesis, which involves multiple steps, or by solvent extraction from non-microbial sources. However, the complex structures of many carotenoids make chemical synthesis challenging and often ineffective. In contrast, microbial manufacturing offers a sustainable alternative and has the potential to meet the increasing need for natural carotenoids. A more sustainable method of producing carotenoids has enabled more sustainable carotenoid production by developments in metabolic engineering and synthetic biology (Saini and Keum 2019; Igreja et al. 2021).

### Engineered strains for enhanced carotenoid production

Β-carotene, astaxanthin, canthaxanthin, zeaxanthin, lutein, and lycopene are the main carotenoids that are in high demand worldwide (Steven et al. 2022; Ashokkumar et al. 2023). Although carotenoid production is industrially valuable, increased pigment accumulation reflects redistribution of fixed carbon rather than an increase in total carbon assimilation capacity. Among these, microalgae are widely used for the production of astaxanthin, β-carotene, and lutein since they usually collect significantly more carotenoids than bacteria or yeast. Microalgae continue to be the principal source for commercial-scale carotenoid production, despite ongoing research and development of microbial production systems based on bacteria and fungus (Gervasi et al. 2020).

Several studies have demonstrated that CRISPR-Cas9 tools may effectively modify carotenoid pathways in a variety of microalgal species. Bai et al. (2022) demonstrated that CRISPR-Cas9 can disrupt several genes at once, resulting in *P. tricornutum* mutants devoid of zeaxanthin epoxidase (ZEP1) and violaxanthin de-epoxidase (VDL2). These modifications clarified the roles of both enzymes in xanthophyll cycle-associated fucoxanthin biosynthesis. In another study, the regulatory role of the blue light-responsive protein Cryptochrome P (CryP) was examined. The researchers found a significant increase in fucoxanthin accumulation and the quantity of fucoxanthin chlorophyll a/c-binding proteins (FCPs) in comparison to the wild type after using CRISPR-Cas 9 to remove the *CryP* gene in *P. tricornutum* (Yang et al. 2022). This suggests the regulatory role of CryP in light-responsive pigment metabolism.

Further advancements were documented in *C. reinhardtii*, where highly effective deletion of the zeaxanthin epoxidase (ZEP) gene was made possible by Cas9 ribonucleoprotein (RNP)-mediated editing. When compared to the wild type, this mutation produces a 56-fold increase in zeaxanthin content and a 47-fold increase in productivity (Grama et al. 2022; Hu et al. 2023b).

In addition to experimentally validated targets, several additional carotenoid biosynthesis genes have been proposed as potential engineering candidates for improving the production of high-value pigment (HVP). Genes such as *bkh* (β-carotene hydroxylase), *psy* (phytoene synthase), *lcy* (Lin et al. (2022) cyclase), and *pds* (phytoene desaturase) play central roles in carotenoid biosynthesis. In species such as *Haematococcus pluvialis,* one of the most commercially important microalgae for natural astaxanthin production, overexpression of targeted modification of these genes has been linked to increased carotenoid yields (Sharon-Gojman et al. 2015). However, efficient genome editing in this species remains challenging because of its slow growth, low transformation efficiency, and thick stress-induced cell wall*.* Genome-enabled metabolic engineering studies in *Chromochloris zofingiensis* suggest that this species could serve as an attractive platform for future CRISPR-mediated optimization of carbon allocation and carotenoid biosynthesis (Roth et al. 2017; Yang et al. 2025). As summarized in Table 3, targeted CRISPR/Cas interventions across multiple microalgal species have enabled measurable improvements in pigment accumulation, carbon assimilation, and growth performance.

**Table 3:** Overview of CRISPR-enabled gene knockouts and transcriptional regulation strategies applied in microalgae to enhance pigment production, photosynthetic efficiency, and biomass yield

Microalgal species	Gene (s) engineered	Editing strategy	CRISPR/Cas system	Trait improvement	References
*Chlamydomonas reinhardtii*	PEPC1 (phosphoenol pyruvate carboxylase)	Transcriptional downregulation	CRISPRi	Increased biomass (~ 2.56 g/L) and increased CO_2_ assimilation (~ 1.087 g-CO_2_/g-DCW) compared to the control Overall enhanced growth under mesothermophilic conditions	Lin and Ng (2023)
*Chlorella vulgaris*	Fad3 (omega-3 fatty acid desaturase)	Knockout	Cas9	Engineered strain showed ~ 20% higher biomass compared to WT ~ 46% increase in lipid content	Lin and Ng (2020)
*Chlamydomonas reinhardtii CC125*	Β-carotene hydroxylase (CHYB)	Knockout	CRISPR/Cas9	Maximal cell density under low-light mixotrophic and highlight photoautotrophic conditions Better high-density culture performance	Kim et al. (2025)
*Chlamydomonas reinhardtii-4349*	Zeaxanthin epoxidase (ZEP) and *CpFTSY*	Double Knockout	Cas9	Zeaxanthin content has increased >10-fold Total chlorophyll decreased ~ 3-fold with 2–3× higher chl Improved growth and light use- efficiency under high light	Baek et al. (2016)
*Chlamydomonas reinhardtii-4349*	Zeaxanthin epoxidase (ZEP)	Knockout	Cas9	Fastest growth, highest biomass, and total carotenoid content Accumulated ≥ 50 more zeaxanthin Lutein remained unchanged	Baek et al. (2018)
*Dunaliella salina CCAP19/18*	β-carotene hydroxylse (BCH)	Single gene Knockout	Cas9	2.2-fold increase in β-carotene content Zeaxanthin levels were correspondingly decreased	Hu et al. (2021a)
*Chlamydomonas reinhardtii-4349*	Zeaxanthin epoxidase (ZEP), lycopene epsilon cyclase (LCYE)	Double Knockout	Cas9	Double knockout blocked α-branch carotenoids directed flux to β-carotene and accumulated 7.28 mg/g zeaxanthin Lutein was absent	Song et al. (2020)
*Chlamydomonas reinhardtii-4349*	Zeaxanthin epoxidase (ZEP), lycopene epsilon cyclase (LCYE)	Double Knockout + CHYB over expression	Cas9	Zeaxanthin biosynthesis was enhanced Increased production of carotenoids, 21.68 mg/L, a 190-fold improvement	Jang et al. (2025)
*Phaeodactylum tricornutum*	Cryptochrome gene (CryP)	Knockout	Cas9	Fucoxanthin content increased 1.29-fold FCP proteins also increased, with a prominent golden colour Photosynthetic activity slightly higher, growth unchanged	Yang et al. (2022)
*Chlamydomonas reinhardtii-4349*	Zeaxanthin epoxidase (ZEP), ADP- glucose pyrophosphorylase (AGP)	Double Knockout	Cas9	2-fold increase in triacyl glycerol (TAG) 35% increase in higher zeaxanthin level Optimized further enhanced macular pigment productivity by ~29% Produced pigment enriched microalgal oil	Song et al. (2022)
*Chlamydomonas reinhardtii*	Lycopene ε-cyclase	Knockout	Cas9	Lutein was completely depleted (2.27 to 0 mg/L) Other carotenoids were increased, zeaxanthin 1.9-fold (0.31 to 0.59 mg/L), antheraxanthin 2.25-fold (0.28 to 0.63 mg/L), and 1.8-fold (1.3 to 2.3 mg/L) Overexpression of CrBKT and CrCHYB further enhanced astaxanthin from 0.8 ± 0.3 to 1.8 ± 0.6 mg/L (~ 2.25-fold)	Kneip et al. (2024)

### Microalgae with improved carbon capture and fixation efficiency

It has been demonstrated that the metabolic engineering techniques that alter internal metabolic pathways in microalgae increase their ability to capture carbon (Ugya et al. 2024). By increasing the efficiency of CO_2_ assimilation into biomass, these improvements help mitigate climate change by lowering greenhouse gas emissions. Furthermore, metabolic engineering can enhance the uptake and utilization of nutrients, resulting in faster growth rates and higher biomass yields (Chen et al. 2020). Although many studies report enhanced biomass accumulation, enzyme activity, or metabolite production, direct measurements of long-term CO_2_ sequestration efficiency remain limited, making large-scale impact difficult to assess. Additionally, these treatments enhance the economic viability of microalgae for bioprospecting applications like biofuel generation and pharmaceutical research by promoting the creation of high-value substances, including lipids, proteins, and antioxidants (Banerjee et al. 2016). Optimizing carbon fixation pathways and enhancing photosynthetic efficiency are important metabolic strategies used to increase carbon capture in microalgae. In *Chlamydomonas reinhardtii,* CRISPRi-mediated repression of PEPC1 increased CO_2_ assimilation efficiency from 0.536 to 1.087 g-CO_2_ g-DCW^−1^, along with enhanced biomass, lipid, and lutein production under mixotrophic conditions. However, intrinsic limitations such as RuBisCO inefficiency and metabolic trade-offs still constrain overall CO_2_ sequestration capacity (Lin and Ng 2023). A comparative summary of CRISPR-based modifications influencing carbon fixation, biomass productivity, and metabolite accumulation in microalgae is presented in Table 4.

**Table 4:** CRISPR-based metabolic and regulatory engineering strategies in microalgae for enhancing carbon assimilation, photosynthetic performance, and biomass productivity, highlighting species-specific outcomes, carbon sequestration relevance, and system -level limitations

Species	Engineering strategy	Primary reported outcome	Relevance to CO_2_ sequestration	System-level interpretation /remaining limitation	References
*Chlamydomonas reinhardtii*	CRISPRi repression of PEPC1	Higher biomass accumulation and improved carbon assimilation efficiency	Redirects intracellular carbon toward biomass formation	Improvement mainly reflects better carbon partitioning rather than overcoming RuBisCO’s inherent catalytic limits	Lin and Ng (2023)
	ZEP knockout	Marked increase in zeaxanthin and overall carotenoid content	An enlarged photoprotective pigment pool may enhance stress tolerance	Increased pigment accumulation does not necessarily translate into higher net CO_2_ fixation	Baek et al. (2018)
	ZEP+AGP double knockout	Concurrent rise in TAG and zeaxanthin accumulation	Indicate coordinated redirection of carbon flux toward storage pathways	Enhanced storage metabolism may come at the expense of sustained growth and biomass yield	Baek et al. (2016)
	CHYB knockout	Better growth performance under high light, photoautotrophic conditions	Improved light stress tolerance can indirectly support productivity	Benefits are strongly condition-dependent and may not persist at scale	Kim et al. (2025)
*Nannochloropsis oceanica*	dCas9-VP64-based transcriptional activation	Improved growth and photosynthetic efficiency	Supports better light utilization and regulatory control of photosynthesis	Gains remain within the physiological limits of energy conversion and carbon fixation capacity	Wei et al. (2022)
	Knockout of RuBisCO lysine methyltransferase and chloroplast aldolase	Improved stress tolerance and biomass accumulation	Stress resilience may indirectly support productivity	Improvements are relatively modest compared to theoretical photosynthetic limits	Liang et al. (2024)
*Phaeodactylum tricornutum*	ALB3b disruption	Better photon uses efficiency and light penetration in dense cultures	Improves light distribution in bioreactors	Light-use gains do not necessarily translate into proportional CO_2_ sequestration increases	Nymark et al. (2019)
	CryP knockout	Increased fucoxanthin accumulation	Enhances carotenoid-driven stress protection	Reflects metabolic rerouting rather than direct enhancement of carbon fixation	Yang et al. (2022)
*Dunaliella salina*	β-carotene hydroxylase knockout	Elevated β-carotene accumulation	Boosts secondary metabolite carbon sink	Carbon allocation toward pigments may reduce biomass formation	Hu et al. (2021a)
*Chlorella sorokiniana*	Truncated antenna engineering	Higher biomass productivity in high-density cultures	Improves light utilization efficiency in reactors	Performance remains highly dependent on reactor design and light availability	Lin et al. (2022)

Overall, experimental studies show that CRISPR-based engineering can enhance specific metabolic traits, including biomass accumulation and carotenoid production. However, these improvements are often modest, condition-dependent, and primarily reflect optimization of metabolic pathways rather than a fundamental increase in CO_2_ fixation capacity. Bridging the gap between laboratory-scale findings and industrial-scale carbon sequestration will require integration of genetic engineering with quantitative performance metrics and bioprocess-level considerations.

## Challenges and limitations

CRISPR/Cas-based genome editing has emerged as a powerful tool for engineering microalgae to improve their industrial and biological traits. Although microalgae comprise an estimated 200,000–800,000 species worldwide, only around 50,000 have been formally characterized, and fewer possess fully annotated genomes (Venkatesan et al. 2015). The understanding of their metabolic network and cellular functions remains limited by the lack of genomic data, which ultimately restricts targeted metabolic engineering efforts. In addition to limited genomic resources to increase microalgal photosynthetic efficiency, several real-world obstacles still prevent their widespread use. Furthermore, many species have poor transformation efficiency, particularly those that are not commonly employed in industry. Off-target mutations and the influence of native gene-silencing mechanisms further reduce editing precision (Lee et al. 2023).

The strong cell wall structure of microalgal cells, which significantly restricts macromolecule uptake, is a major obstacle to introducing genetic material into microalgal cells (Wang et al. 2015). Traditional physical and chemical pretreatments, such as ultrasonication, bead milling, high-pressure homogenization, acid or alkaline treatments, and strong oxidants, have been commonly used to improve transformation efficiency (Ren et al. 2022). More recently, zinc oxide nanowire-array-assisted droplet electroporation has shown improved performance in species possessing rigid cell walls (Guihéneuf et al. 2016).

Transformation success is also strongly dependent on the species. For instance, although *Haematococcus lacustris* is an important producer of astaxanthin, its transformation efficiency remains low due to its slow growth and thick stress-induced cell wall (Tran and Kaldenhoff 2020).

CRISPR tool performance also varies markedly between microalgal lineages. SpCas9 remains the preferred nuclease in *Chlamydomonas reinhardtii,* whereas Cas12a variants often provide superior editing efficiency in diatoms such as *Phaeodactylum tricornutum* and in *eustigmatophytes*. Homology-directed repair (HDR) efficiencies are generally very low (< 1%) across most microalgae, although in *C. reinhardtii,* they can be improved to approximately 5–10% using approaches such as NHEJ suppression and cell cycle synchronization. Although dCas9-VP64 performs efficiently in *C. reinhardtii*. However, stronger activator complexes such as VPR or SAM are often required in species including *Haematococcus pluvialis* and *Nannochloropsis gaditana* (Jeon et al. 2017; Lin et al. 2022). Heterologous viral promoters such as SV40 or CMV are effective in certain strains of *Chlamydomonas* but often exhibit limited functionality in diatoms, which further highlights the necessity for species-specific optimization (Bolaños-Martínez et al. 2022; Yang and Lu 2025). The intrinsically low efficiency of homologous recombination makes it difficult to achieve precise knock-ins in microalgae. In species like *Chlamydomonas reinhardtii* (Plecenikova et al. 2013) and *Phaeodactylum* (Moosburner et al. 2020)*,* homologous recombination rates have remained low despite numerous optimization efforts, including changing donor DNA concentrations, altering the length of HDR templates, and fine-tuning transformation parameters. However, Naduthodi et al. (2019) showed that HDR efficiency can be substantially improved by co-introducing a Cas9-gRNA ribonucleoprotein complex with a double-stranded DNA repair template, resulting in a significantly larger fraction of correctly targeted integrants.

Genome editing in biotechnology and agriculture is strictly controlled in many nations, and discussions over its safety and ethical implications are still ongoing. The need to review and update current GMO definitions and regulatory frameworks is developing because of the quick development of editing technologies (Spicer and Molnar 2018). Even micro-algal strains that have had only their endogenous genes altered are categorized as genetically modified organisms (GMOs) in the European Union, which creates obstacles to their practical and commercial applications. Therefore, before the advent of editing technologies, understanding the regulatory frameworks governing gene-edited microalgae is essential for future industrial deployment (Dhokane et al. 2023).

### Reactor-scale constraints and bioprocess limitations

Although CRISPR/Cas technology has significantly improved carotenoid production in microalgae, translating these laboratory-scale successes to an industrial-scale cultivation remains challenging. Engineered strains that perform well under controlled laboratory conditions often show reduced stability and productivity when exposed to the complex environmental conditions of large-scale photobioreactors.

One of the major limitations is light penetration and self-shading in dense cultures. CRISPR-engineered strains with reduced light-harvesting antenna systems can improve light distribution and enhance photosynthetic efficiency. However, lowering protective pigment content may also increase sensitivity to photoinhibition and oxidative stress under fluctuating or excessive light conditions (Cano et al. 2023; Kneip et al. 2024). Therefore, careful optimization of light intensity, reactor configuration, and cultivation conditions is essential during scale-up.

Gas exchange is another important challenge in a large-scale system. Enhanced photosynthetic activity in engineered strains can lead to oxygen accumulation, which may inhibit cell growth and reduce overall productivity if not efficiently removed. At the same time, high carotenoid-producing strains require a continuous supply of CO_2_ to maintain carbon fixation and metabolite synthesis (Nazari et al. 2024; Perfeito et al. 2026).

Large bioreactors also experience temperature, nutrient, and pH gradients because mixing becomes less efficient at scale. These fluctuations can negatively affect engineered metabolic pathways and reduce carotenoid accumulation. In addition, some CRISPR modifications targeting cell wall biosynthesis may improve downstream extraction efficiency, but they can also make cells more sensitive to shear stress generated during mixing and aeration (Yu et al. 2024).

Contamination and biosafety concerns further complicated industrial cultivation. Outdoor and large-scale systems are particularly vulnerable to microbial contamination, while genetically engineered microalgae require straight containment measures and regulatory approval. Moreover, downstream processing remains expensive because carotenoids are intracellular compounds that require efficient cell disruption and extraction technologies (Li et al. 2026).

However, most studies are still limited to shaking flasks and photobioreactors, and there is notable lack of pilot or industrial scale validation. Scale-down studies indicate that real-world factors like light gradient, gas transfer, and mixing can strongly affect performance. As a result, current yield values should be treated as ideal upper limits, and more scale-relevant studies are needed to confirm their true industrial potential.

## Future perspectives and research directions

The development of an economically viable large-scale production system is supported by CRISPR-based technologies, which provide integrated strategies to enhance photosynthetic performance and biomass yield (Jiang et al. 2025; Mutale-joan and El Arroussi 2025; Wang et al. 2025). CRISPR interference (CRISPRi) can be used to precisely modulate gene expression to enhance photosynthesis (Patel et al. 2019). By introducing targeted point mutations in genes like carbonic anhydrases and bicarbonate transporters, base editing further strengthens CCMs and improves CO_2_ affinity under industrial conditions. These approaches can be combined with multiplexed CRISPRi to suppress competing carbon utilization pathways (Li et al. 2023). Further advancements include base/prime editing to fine-tune carbon flow regulators like PEPC for improved biomass accumulation, CRISPR activation (CRISPRa) to optimize engineered bypass pathways, and CRISPRi-mediated reduction of photorespiratory enzyme activity to reduce photorespiratory carbon loss. While CRISPRa encourages quicker NPQ relaxation, base or prime editing of NPQ-related proteins can also improve photoprotection by altering their response kinetics (Hu et al. 2023b).

CRISPR provides coordinated control over lipid metabolism to improve biofuel yield beyond photosynthetic optimization. Integrated CRISPRa/i and precision-editing strategies may enable coordinated regulation of lipid biosynthesis, fatty acid composition, and TAG turnover (Goswami et al. 2022). Real-time regulation of lipid induction is also made possible by emerging CRISPR-based biosensors and optogenetic circuits (Antonacci and Scognamiglio 2020; Patwari et al. 2023). Successful applications will require extending the technologies to a wider variety of commercially valuable species while guaranteeing genetic stability, scalable production, and adherence to changing regulatory frameworks. Comprehensive evaluations of environmental safety and economic viability, including ecological risk assessment and effective biocontainment techniques, are equally crucial (Yang and Lu 2025). Looking ahead, research in this area is shifting toward more sophisticated methods such as multiplex genome editing and the incorporation of synthetic biology tools, along with increasing emphasis on enabling efficient genetic engineering of commercially important non-model algal species (Seralathan et al. 2025).

### Rational selection of multifunctional target genes for multi-trait engineering

Rational selection of CRISPR targets should move beyond single-trait genes to regulatory nodes that coordinate photosynthetic efficiency, biomass accumulation, stress tolerance, and carotenoid production. Multi-omics integration of genomics, transcriptomics, proteomics, and metabolomics, together with gene regulatory network and metabolic-flux analyses, could provide a systematic framework for identifying candidate genes that connect carbon assimilation, photosynthetic performance, carbon partitioning, stress responses, and pigment metabolism (Mishra et al. 2019; Kuo et al. 2022). Candidate targets could be prioritized based on consistent relationships among gene expression, protein activity, metabolite profiles, and multiple phenotypic traits across relevant growth and stress conditions, rather than relying solely on differential expression from a single omics dataset.

Before CRISPR intervention, systematic molecular and physiological characterization could help determine the pathway connectivity of candidate genes and predict potential metabolic trade-offs. For example, genes involved in carbon partitioning, light harvesting, and CCM regulation could be evaluated for their potential effects on carbon allocation, energy balance, growth, and stress responses. Such pre-editing analysis may help identify targets that improve one trait without compromising other desirable cellular functions and thereby enable more rational and predictable genome engineering (Kuo et al. 2022).

Several gene classes may therefore represent promising candidates for multifunctional engineering, including carbon partitioning regulators such as PEPC1, light harvesting components, CCM-associated genes, and stress response regulators (Kao and Ng 2017; Lin and Ng 2023). However, experimentally validated microalgal hub genes capable of simultaneously improving photosynthetic efficiency, biomass productivity, stress tolerance, and carotenoid synthesis remain limited. Future studies should therefore combine multi-omics guided target prioritization with CRISPR-based functional screening, metabolic flux analysis, and quantitative phenotyping to identify and validate dual-function or hub regulators for coordinated multi-trait engineering.

Recent advances in artificial intelligence (AI) could further strengthen this target selection framework by integrating multi-omics datasets and predicting cis-regulatory elements, enzyme properties, beneficial protein mutations, protein–protein interactions, promoter strength, and CRISPR guide performance. A systematic molecular framework integrating chromatin state and epigenetic information with transcription-factor binding, protein regulation, enzyme activity, metabolic flux, and carotenoid sequestration could help prioritize mechanistically relevant targets such as DXS, PSY, PDS, LYCB, BKT, and BCH rather than relying solely on differential expression (Hu et al. 2021b; Zhao et al. 2024; Liang et al. 2026). Within a design-build-test-learn (DBTL) cycle, genome-scale metabolic models and machine-learning approaches could guide strain design, while genome editing and synthetic regulatory elements could implement predicted modifications. However, limited algal-specific datasets, model interpretability, cross-species generalization, and the need for experimental validation remain important challenges (Liang et al. 2026).

Transcription factors (TFs) represent another promising class of multifunctional CRISPR targets. Studies in higher plants have demonstrated that editing TFs can simultaneously improve productivity and stress- or pigment-related traits, illustrating the potential of regulatory nodes for multi-trait engineering (Sugano and Nishihama 2018). However, systematic identification and validation of TFs that coordinate photosynthetic efficiency, biomass accumulation, stress tolerance, and carotenoid biosynthesis remain limited in microalgae. Future studies should therefore prioritize genome-wide identification of candidate TFs through transcriptomic, chromatin-accessibility, and regulatory network analyses, followed by CRISPR knockout or CRISPRi/CRISPRa screening under contrasting growth and stress conditions (Sagharyan et al. 2025). Functional validation of these candidates using quantitative measurements of photosynthesis, biomass, stress responses, carbon partitioning, and carotenoid accumulation could reveal regulatory hubs capable of coordinating multiple desirable traits while minimizing the need for independent editing of multiple pathway genes.

## Conclusions

CRISPR-based genome engineering has emerged as a powerful tool for improving microalgae as a sustainable platform for CO_2_ sequestration and high-value carotenoid production. Recent studies show that targeted gene knockouts, site-specific integrations, CRISPRi/CRISPRa systems, and multiplex genome editing can effectively modify photosynthetic pathways, carbon concentrating mechanisms, and carotenoid biosynthesis. These strategies mainly improve how carbon is distributed and utilized within the cell, rather than overcoming the fundamental biological limitations of photosynthesis itself. Research in model organisms such as *Chlamydomonas reinhardtii* has provided valuable mechanistic insights and engineering strategies that are now being explored in more industrially relevant microalgal species. Unlike established heterotrophic platforms such as *E. coli* and *S. cerevisiae,* which require organic carbon substrates and lack native photosynthetic CO_2_ fixation, microalgae can photosynthetically fix CO_2_ without direct dependence on arable land and naturally synthesize valuable carotenoids and lipids. These distinctive features justify continued development of efficient genetic tools for microalgae to exploit their native photosynthetic and metabolic capabilities for sustainable carbon sequestration and bioproduct production.

However, several important challenges still limit the large-scale application of CRISPR-engineered microalgae. Transformation efficiency remains highly dependent on species and delivery method; homologous recombination is often inefficient, and concerns related to off-target effects and regulatory approval continue to restrict practical deployment. In addition, RuBisCO remains a major bottleneck because of its slow catalytic activity and oxygenase function, while the carbon concentrating mechanism requires substantial energy input, creating trade-offs between enhanced CO_2_ fixation and cellular energy balance. Many published studies also focus mainly on increases in biomass or metabolite accumulation, whereas quantitative evaluations of CO_2_ fixation rates, carbon conversion efficiency, and long-term sequestration performance are still limited. Furthermore, translating laboratory-scale improvements to industrial cultivation remains challenging because of additional constraints such as light penetration, CO_2_ mass transfer, mixing efficiency, and culture stability.

Future progress will depend on combining CRISPR-based engineering with systems biology, quantitative metabolic analysis, and bioprocess optimization. Advances in delivery technologies, expanded CRISPR toolkits, multi-omics approaches, machine learning-guided strain design, and synthetic regulatory systems may further improve the precision and stability of microalgal engineering. However, meaningful improvements in carbon sequestration will likely require integrated solutions that address both biological and reactor-scale limitations. Overall, CRISPR-enabled microalgal engineering offers significant potential for supporting sustainable carbon management and high-value bioproduct generation within a circular bioeconomy.

## Data Availability

Data will be made available on request.
